# The acromioclavicular ligament shows an early and dynamic healing response following acute traumatic rupture

**DOI:** 10.1186/s12891-020-03614-6

**Published:** 2020-09-04

**Authors:** Dirk Maier, Lars-Rene Tuecking, Anke Bernstein, Gernot Lang, Ferdinand Christian Wagner, Martin Jaeger, Peter Ogon, Norbert Paul Südkamp, Kaywan Izadpanah

**Affiliations:** 1 Department of Orthopaedics and Trauma Surgery, Medical Center – University of Freiburg, Faculty of Medicine, University of Freiburg, Hugstetter Str. 55, 79106 Freiburg, Germany; 2 Department of Orthopaedic Surgery, Medical School Hannover, Diakovere Annastift, Anna-von-Borries-Str. 1-7, 30625 Hannover, Germany; 3 Section Musculoskeletal Biomaterials of G.E.R.N. Research Center, Department of Orthopaedics and Trauma Surgery, Medical Center – University of Freiburg, Faculty of Medicine, University of Freiburg, Hugstetter Str. 55, 79106 Freiburg, Germany; 4 Center of Orthopaedic Sports Medicine Freiburg, Breisacher Str. 84, 79110 Freiburg, Germany

## Abstract

**Purpose:**

Symptomatic horizontal instability is clinically relevant following acute acromioclavicular joint dislocations. However, the intrinsic healing response is poorly understood. The present study sought to investigate time-dependent healing responses of the human acromioclavicular ligament following acute traumatic rupture.

**Methods:**

Biopsies of the acromioclavicular ligament were obtained from patients undergoing surgical treatment for acute acromioclavicular joint dislocations. Specimens were stratified by time between trauma and surgery: group 1, 0–7 days (*n* = 5); group 2, 8–14 days (*n* = 6); and group 3, 15–21 days (*n* = 4). Time-dependent changes in cellularity, collagen (type 1 and 3) concentration, and histomorphological appearance were evaluated for the rupture and intact zone of the acromioclavicular ligament.

**Results:**

Group 1 was characterized by cellular activation and early inflammatory response. The rupture zone exhibited a significantly higher count of CD68-positive cells than the intact zone (15.2 vs 7.4; *P* ≤ 0.05). Consistently, synovialization of the rupture end was observed. Within the second week, the rupture zone was subject to proliferation showing more fibroblast-like cells than the intact zone (66.8 vs 43.8; *P* ≤ 0.05) and a peak of collagen type 3 expression (group 1: 2.2 ± 0.38, group 2: 3.2 ± 0.18, group 3: 2.8 ± 0.57; P ≤ 0.05). Signs of consolidation and early remodeling were seen in the third week.

**Conclusions:**

The acromioclavicular ligament exhibits early and dynamic healing responses following acute traumatic rupture. Our histological findings suggest that surgical treatment of acute ACJ dislocations should be performed as early as possible within a timeframe of 1 week after trauma to exploit the utmost biological healing potential. Prospective clinical studies are warranted to investigate whether early surgical treatment of ACJ dislocations translates into clinical benefits.

## Introduction

Acromioclavicular joint (ACJ) dislocations account for 3–12% of shoulder girdle injuries and occur with an incidence of about 5/100,000 individuals per year [1]. The optimal therapeutic management has been controversially discussed over the past decades. In most European countries, acute high-grade ACJ injuries (Rockwood type IV and V) are routinely treated surgically, whereas non-operative therapeutic approaches are common in Northern America [2, 3]. According to a survey among orthopedic surgeons in the United States, the term ‘acute’ was defined as a timeframe of less than 3 weeks after injury [4]. On the contrary, a recent study defined the term ‘acute’ as < 6 months which reflects the major controversies on the definition and timing of operative treatment [5]. In a French multicenter study, Barth et al. found superior radiological restoration of the coracoclavicular distance if surgery was performed within 10 days after injury [6]. The authors concluded that surgery performed within the early inflammatory phase has beneficial effects due to the superior ligamentous healing potential compared to delayed treatment. Though, profound evidence supporting early surgical treatment in ACJ dislocations based on experimental studies on the intrinsic healing response is lacking. Despite technical advancements in acute ACJ stabilization including acromioclavicular reconstruction and cerclage, persistent dynamic horizontal instability (DHI) still reflects a clinically relevant issue [7–9]. Symptomatic DHI occurs in 50% following acute ACJ stabilization and can only be partially addressed by additional acromioclavicular stabilization techniques [7, 10–12]. Recent biomechanical studies showed that the anatomical repair of the entire superior acromioclavicular ligament complex (ACLC) may restore posterior and rotational stability of the ACJ [13, 14]. Though, methodological limitations of experimental biomechanical cadaveric studies have to be considered. Causatively, insufficient intrinsic healing of the acromioclavicular ligament complex can be assumed as a major cause of persistent symptomatic DHI. Additional causes may be technical failures (e.g. malposition/widening of bone tunnels) [14] or implant failure (dislocation/breakage, peri-implant fracture) [15, 16]. Histomorphological studies of the anterior cruciate ligament (ACL) and medial collateral ligament (MCL) of the human knee joint described specific overlapping phases of healing (inflammation, proliferation, and remodeling) and found fundamental differences between intraarticular and extraarticular ligaments [17–24]. In a rabbit model, Menetrey et al. described a superior healing potential of MCL tears compared to ACL tears [22]. The authors found a significant increase of αSMA-positive myofibroblasts within 3 days after injury in MCL tears, suggesting the existence of an early dynamic healing response. Correspondingly, the human ACLC as an anatomically comparable periarticular ligament could exhibit similar dynamic healing responses.

The purpose of this experimental study was to investigate time-dependent histological changes of the human ACLC following acute traumatic rupture. Specifically, we wanted to evaluate whether early surgical intervention may be associated with intrinsic biological benefits in operative treatment of acute ACJ dislocations.

## Methods

### Study population

Between January 2014 and July 2016, a prospective, experimental single-center study was conducted in patients undergoing surgical treatment for acute-traumatic ACJ dislocations utilizing hook plate stabilization. The study was approved by the local institutional review board (protocol number 490/13) and informed consent was obtained from all patients before surgery and study participation. Inclusion criteria were age ≥ 18 and ≤ 60 years and presence of an isolated acute traumatic, full-thickness ACJ dislocation (Rockwood type 4 or 5). Patients with (1) chronic ACJ dislocations (> 21 days after trauma), (2) radiological signs of ACJ osteoarthritis, (3) history of previous ACJ injury or surgery and/or (4) comorbidities with assumable impairment of ligament healing (e.g. malignancies, immunosuppression, diabetes) were excluded. Indication for surgery was not influenced by the patient’s decision on study participation. During the study period, 15/64 (23%) of eligible patients were included. Forty nine patients were excluded as they refused to participate in the study (*n* = 33) or lacked detailed documentation (*n* = 16).

Patients were stratified according to the time interval between trauma and surgery: group 1 (< 7 days; *n* = 5), group 2 (8–14 days; *n* = 6), and group 3 (15–21 days; *n* = 4).

### Surgery and biopsy

A coronal slice biopsy of the posterior aspect of the superior ACLC was harvested according to a standardized protocol during surgery. All surgeries were performed by two board-certified orthopedic surgeons (D.M. and K.I.). Mini-open anatomic ACJ reduction and hook plate stabilization were conducted as described previously [25]. A 2-mm-thick coronal slice biopsy involving the entire medial-to-lateral extension of the ACLC was harvested from its posterior aspect using a scalpel. The superior part of the intact insertion zone was marked with a tracking suture to ensure reliable identification of the intact zone (IZ) and rupture zone (RZ) during histological assessment. All biopsies were immediately submitted to the laboratory for freezing and cryosectioning.

### Histology

Tissue samples were immersed in isopentane and embedded in Tissue Tek, O.C.T.™ compound (Sakura Finetek Europe B.V., Alphen aan den Rijn, Netherlands) for flash freezing. Hereafter, samples were cut with Cryostat Leica CM3050 S (Leica Biosystems Nussloch GmbH, Nussloch, Germany) into 7-μm-thick slices that were transferred to microscopic slides (Carl Roth, Karlsruhe, Germany) and stored at − 80 °C until staining. Giemsa staining was used to evaluate cellularity, cell morphology, and extracellular matrix configuration.

### Immunohistochemistry

Immunohistochemistry (IHC) was performed using the ZytoCHEM-Plus HRP Polymer-Kit (ZYTOMED Systems, Berlin, Germany). Initially, sections were fixed with formalin (Roti®-Histofix 4%, Carl Roth, Germany), followed by blocking of endogenous peroxidase activity by incubation with 3% hydrogen peroxide (Carl Roth GmbH + Co. KG, Karlsruhe, Germany). Tissue sections were then incubated overnight with mouse monoclonal or rabbit polyclonal antibodies against the myofibroblast marker alpha-smooth muscle actin (α-SMA), (Abcam; Cambridge, United Kingdom; ab7817; 1:100); the macrophage marker CD68 (DakoCytomation, Glostrup, Denmark; M0718; 1:50); collagen type 1 (Abcam; ab6308; 1:100); and collagen type 3 (Abcam; ab7778; 1:500). The AEC Substrate Kit (ZYTOMED Systems, Berlin, Germany) was then used for visualization of HRP activity (bound antibody complex) in brown-red color. After immunostaining, sections were counterstained with hematoxylin (Haematoxylin QS, VECTOR, Germany) and mounted in Faramount Aqueous Mounting Medium (DakoCytomation, Glostrup, Denmark).

### Digital image analysis

Images were captured with a camera-assisted Olympus X53 microscope (camera model UC50) using OLYMPUS Stream Image Analysis Software 1.9 (Olympus Corporation, Tokyo, Japan). Within each specimen, three high power fields (HPFs) with a magnification of 400× per staining were randomly chosen within the RZ and intact insertion zone IZ of the ACLC. Analysis of the intact ACLC insertion zone (control) served as an intra-individual reference for the evaluation of time-dependent histological changes within the RZ. An experienced expert in musculoskeletal histology (A.B.) performed a descriptive analysis of histomorphological changes. Cellular analysis was simultaneously carried out by three investigators (D.M., L.T., and K.I.) following a consensus principle and consisted of the total cell count, and the counts of αSMA-positive (myofibroblasts), CD68-positive (inflammatory cells), and fibroblast- and fibrocyte-like cells. Fibroblast-like cells were defined as cells exhibiting a nuclear aspect ratio (NAR) ≥5, whereas cells with an NAR < 5 were considered as fusiform, fibrocyte-like cells [26, 27]. Total collagen type 1 and 3 contents were evaluated with the modified Remmele score consisting of a 5-point-scale semi-quantitative score (range, 0–4) [24]. A score of 0 was indicative of no collagen content (negative control), while a score of 4 corresponded to a maximum amount of collagen (positive control). Polarized light microscopy was used for crimp length analysis. Crimp length was measured in μm as the distance between crimp wave apexes (OLYMPUS Stream Image Analysis Software 1.9). Any interobserver differences were resolved by consensus decision.

### Statistical analysis

Statistical analysis was performed using SPSS v22 (IBM, Armonk, NY, USA). Descriptive results are given as mean values with ranges or standard deviations (± SD). Normality of data was tested using the Kolmogorov–Smirnov test. Normally distributed data were analyzed with two-way factorial ANOVA to determine statistically significant intergroup differences. Intragroup differences (IZ vs RZ) were analyzed with the paired *t*-test. Intergroup demographic parameters were compared with chi-square testing. Significance levels were set at two-tailed *P* < 0.05 with Bonferroni post-hoc testing to adjust for multiple group comparisons.

## Results

### Baseline characteristics

**Fig. 1:**
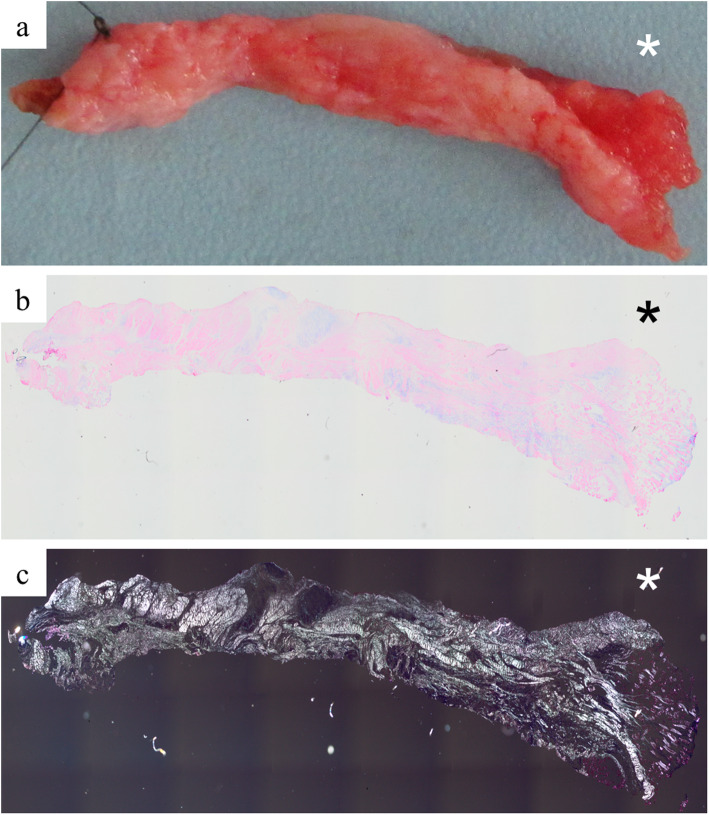
Biopsy of the superior ACLC harvested from a patient 5 days after injury (group 1). Asterisks indicate the rupture zone. **a** macroscopic aspect of the entire biopsy. **b** Giemsa staining. **c** polarized light microscopy

Fifteen patients (1 woman, 14 men) with acute-traumatic ACJ dislocations (Rockwood type 4 or 5) were included in the study (mean age: 37.3 ± 4.56 years; range: 19–54 years; Table 1). Trauma was induced by bicycle and motorbike accidents as wells as high-impact, sports-associated falls onto the lateral shoulder girdle. Patients were assigned to three groups according to time intervals between trauma and surgery (Table 1). Intraoperatively placed tagging sutures allowed for reliable identification of the IZ and RZ. Localizations of all ACLC rupture sites could be confirmed histomorphologically by Giemsa staining and polarized light crimp analysis (Fig. 1).

**Table 1:** Demographic characteristics of the study population

	Group 1 (0–7 days)	Group 2 (8–14 days)	Group 3 (15–21 days)	*P* value
Group size (n)	5	6	4	
Mean age (years±SD)	31.6 ± 11.2	37.7 ± 12.6	42.8 ± 8.1	0.402
Sex				
Female (n)	0	1	0	0.447
Male (n)	5	5	4	
Injured side				
Left (n)	2	1	2	0.509
Right (n)	3	5	2	
Rockwood classification				
Rockwood 4 (n)	1	1	3	0.118
Rockwood 5 (n)	4	5	1	

*SD* Standard deviation

### Time-dependent healing response

#### Cellularity

The total cell count (cells/HPF) in the RZ increased significantly from group 1 (48.1/HPF) to group 3 (149.7/HPF; *P* ≤ 0.05). Additionally, there were significant differences in total cell count between the RZ and IZ groups in group 2 (58.4/HPF vs 83.3/HPF; *P* < 0.05) and group 3 (90.7/HPF vs 149.7/HPF) (*P* < 0.05).

#### Fibroblasts

**Fig. 2:**
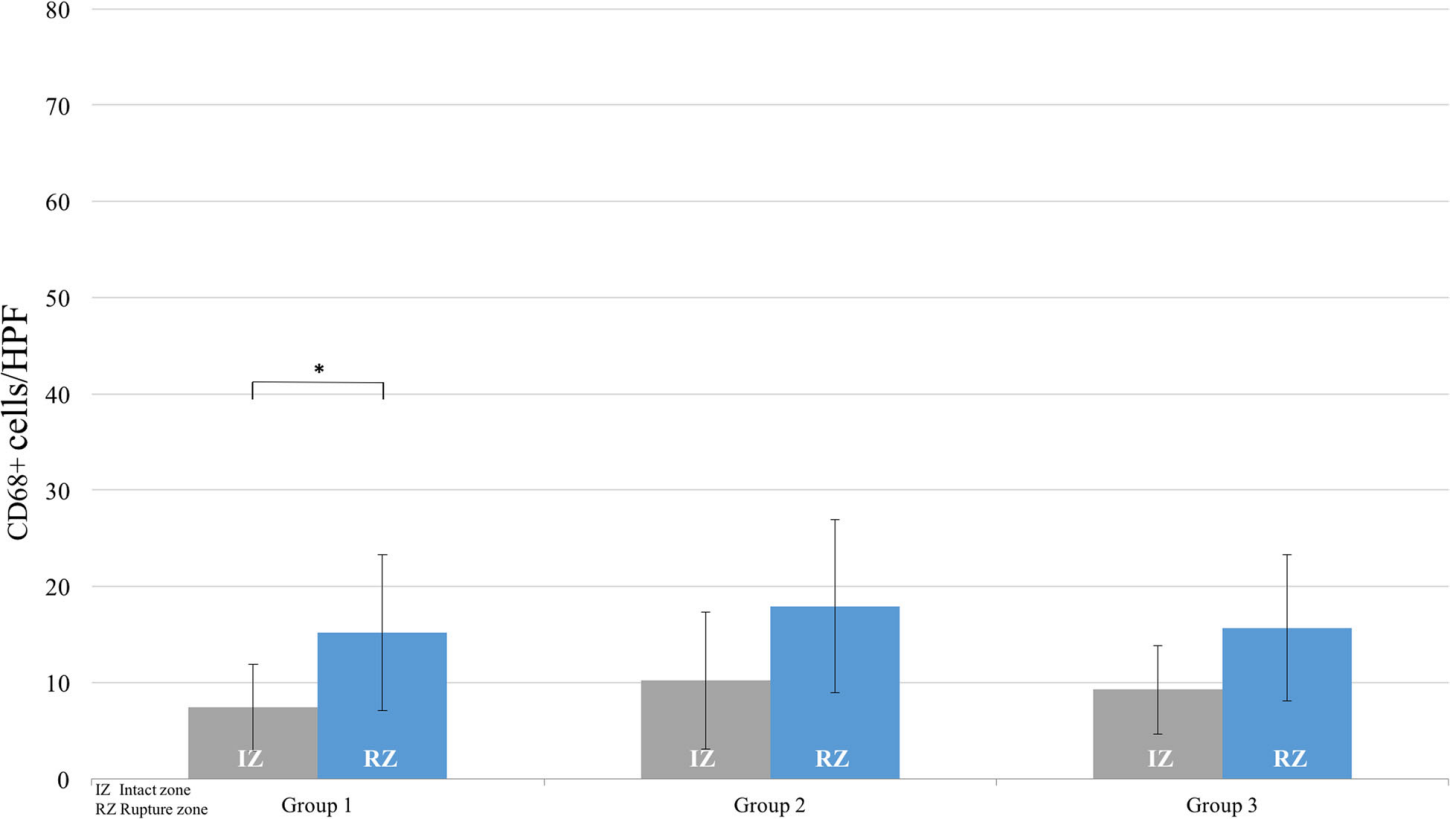
Time-dependent changes in the cellularity of fibroblast-like cells

The total count of fibroblast-like cells in the RZ indicated a peak of group 3 within the third week (Fig. 2). A significant cell count difference was observed between the RZ of group 1 and group 3 (32.5/HPF vs 107.8; *P* < 0.05). Additionally, significant intragroup differences between RZ and IZ were found in groups 2 and 3 (group 2: 66.8/HPF vs 43.8/HPF, group 3: 107.8/HPF vs 50.8/HPF; P < 0.05).

#### αSMA-positive cells

**Fig. 3:**
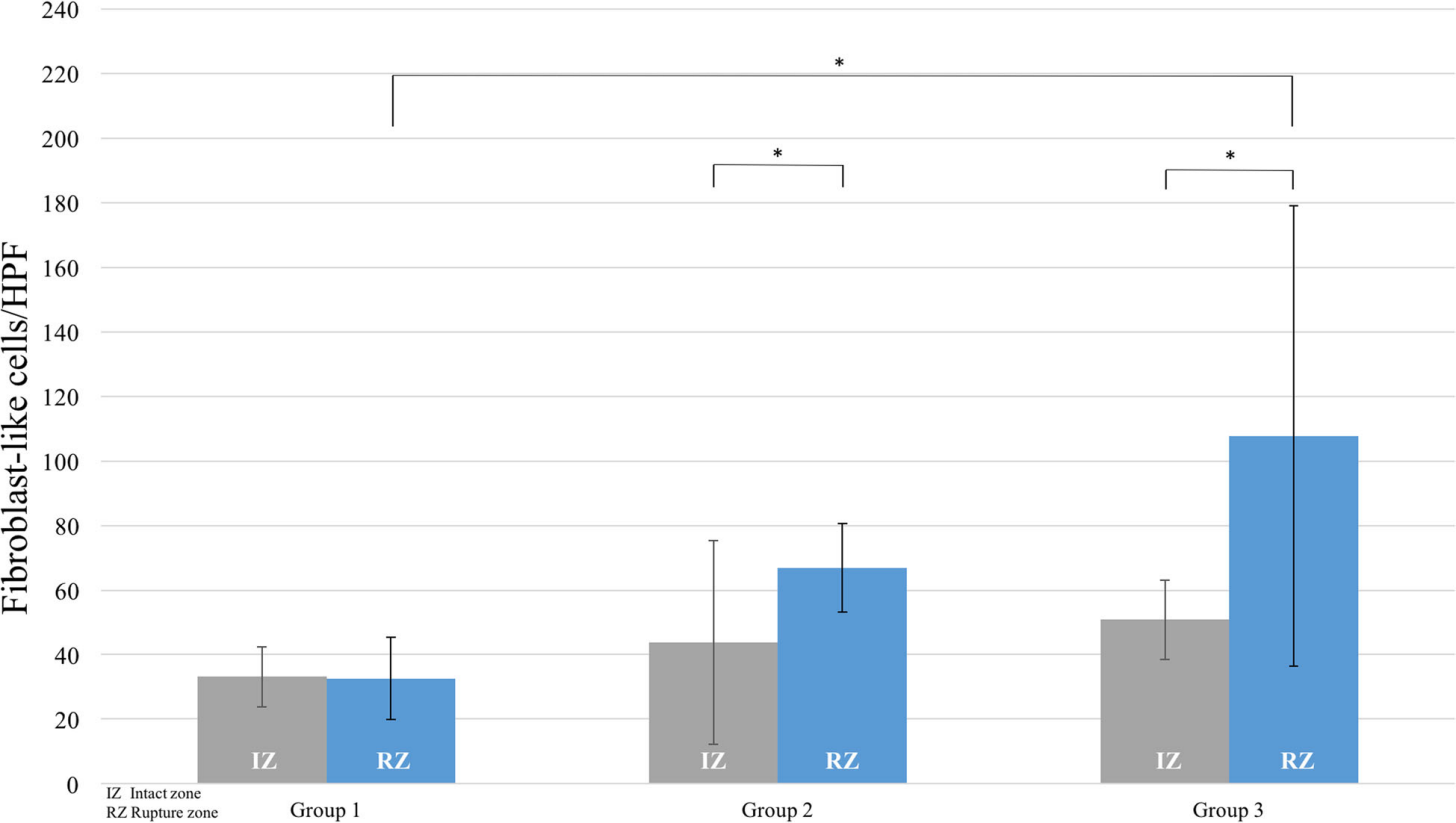
Time-dependent changes in the cellularity of αSMA-positive cells

**Fig. 4:**
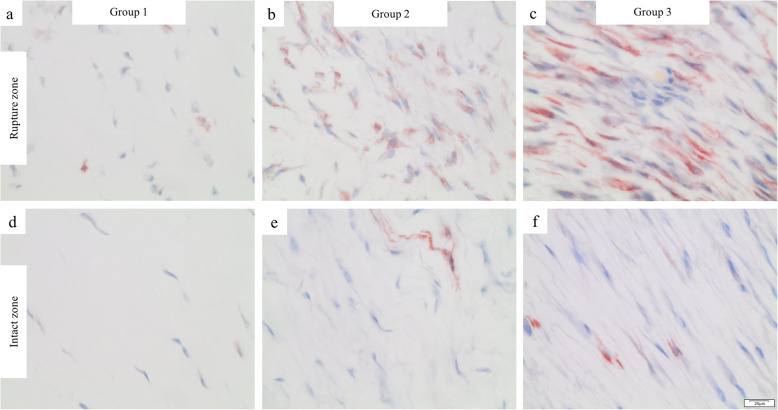
Immunohistochemical assessment of αSMA-positive cells. Rupture zones: **a** (group 1), **b** (group 2), **c** and (group 3). Intact zones: **d** (group 1), **e** (group 2), **f** (group 3). Magnification: 400×, scale bars: 20 μm

The total cell count in the RZ increased from week 1 to week 3 after the trauma, however, this difference was not statistically significant (*p* = 0.074; Fig. 3). Qualitative assessment of αSMA-positive cells utilizing IHC confirmed the time-dependent increase (Fig. 4).

#### CD68-positive cells

**Fig. 5:**
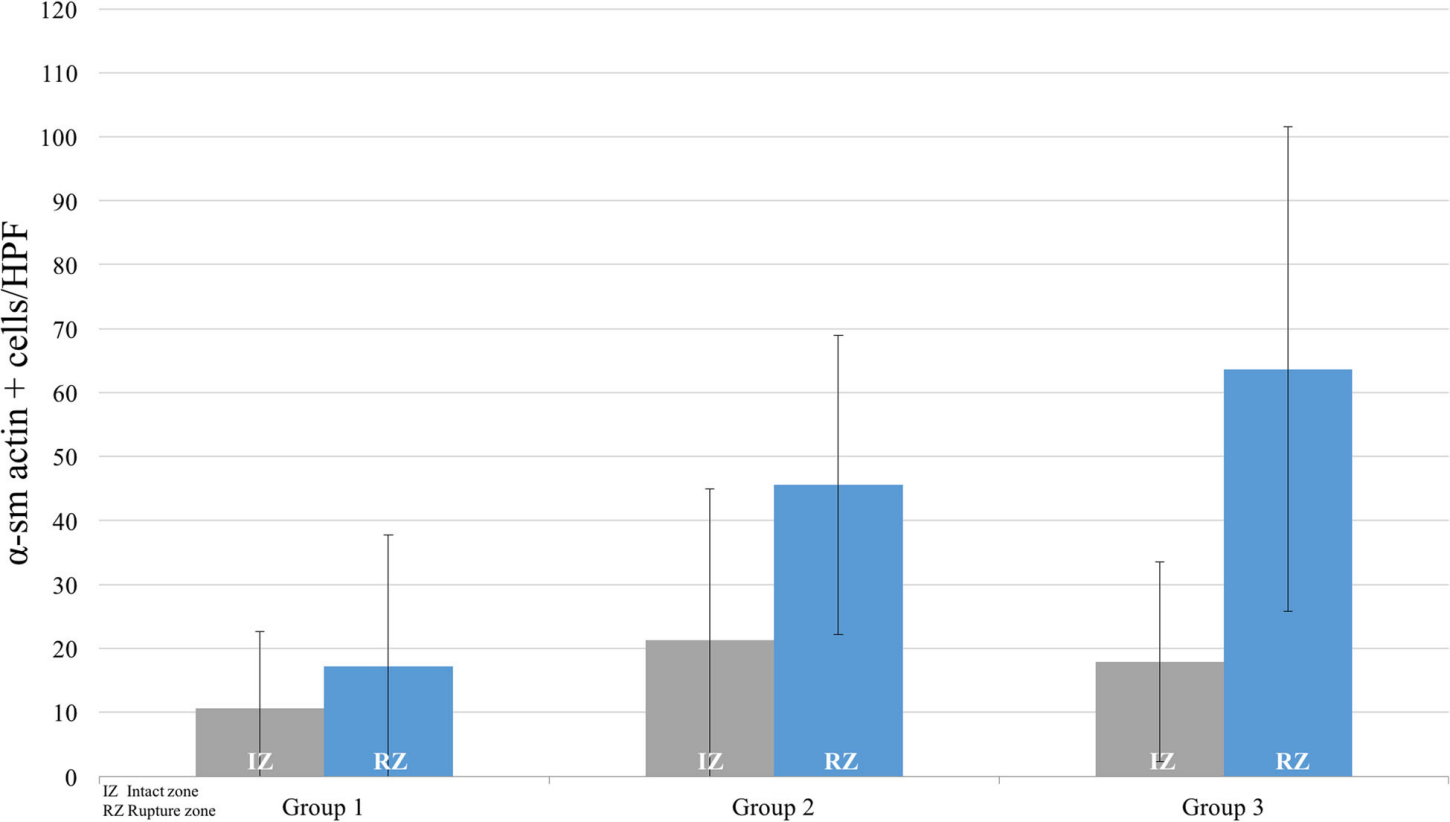
Time-dependent changes in the cellularity of CD68-positive cells

Total cell counts in the RZ did not significantly differ among the three groups (Fig. 5; group 1: 15.2/HPF, group 2: 17.9/HPF, group 3: 15.6/HPF). However, in the first week after trauma, an already significantly higher count of CD68-positive cells was seen in the RZ compared to the IZ (15.2/HPF vs 7.4/HPF; *P* ≤ 0.05). Over time, the proportion of CD68-positive cells in the RZ significantly decreased from 36.7% (group 1) to 12.7% (group 3; *P* < 0.05). Cell counts within the IZ were lower than in the RZ in all investigated biopsies (Fig. 5).

#### Collagen content

**Fig. 6:**
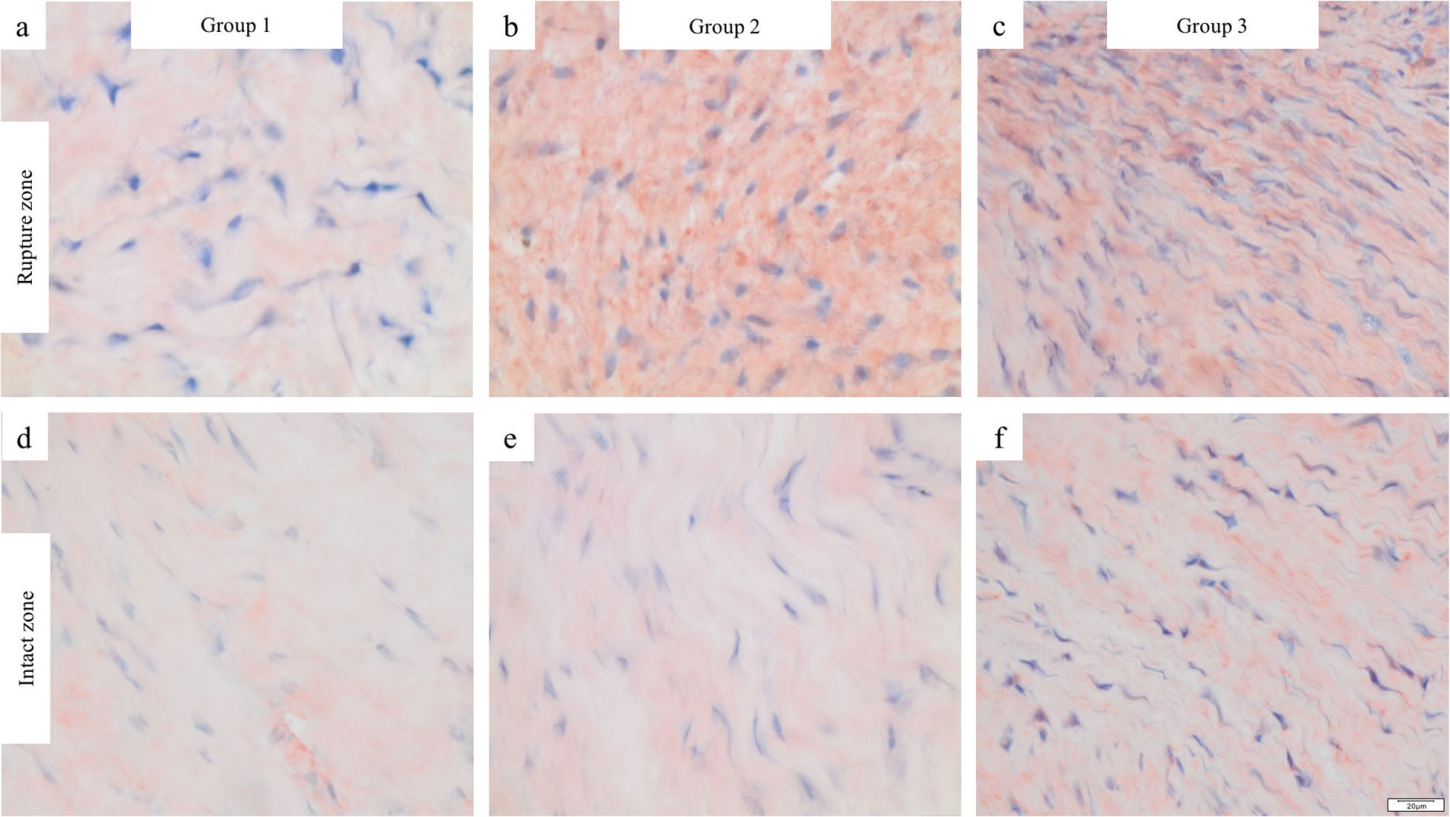
Immunohistochemical assessment of collagen 3 content. Rupture zones: **a** (group 1), **b** (group 2), **c** (group 3). Intact zones: **d** (group 1), **e** (group 2), **f** (group 3). Magnification: 400×, scale bars: 20 μm

The semi-quantitative assessment of collagen 3 content (modified Remmele score) within the RZ was 2.2 ± 0.38, 3.2 ± 0.18, and 2.8 ± 0.57 in groups 1, 2, and 3, respectively (Fig. 6). Within the first week after trauma, an already marked increase of collagen 3 within the RZ compared to the IZ (Fig. 6a, d) was observed. The RZ of group 2 (2 weeks after trauma) comprised the highest collagen 3 content (Fig. 6e) within the entire timeframe of 3 posttraumatic weeks (*P* < 0.05). In group 3, the collagen 3 content slightly decreased within the RZ, and parallel reorganization of collagen fibers was observed (Fig. 6f). Intergroup comparison of collagen 3 contents of the IZ showed a time-dependent increase (*P* > 0.05) (Fig. 6a, b, c). Contents of collagen 1 were stable throughout the period of 3 weeks in both the RZ and IZ. However, a trend for a time-dependent decrease of collagen 1 was observed for IZ across all three groups.

### Histomorphology

#### Rupture zone

**Fig. 7:**
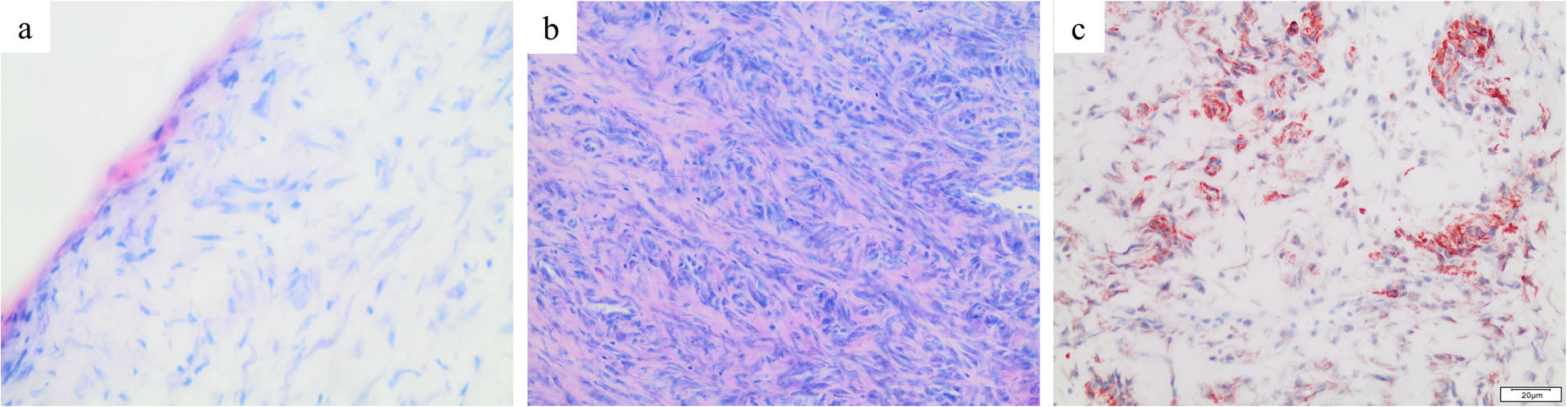
Time-dependent histomorphological changes within the rupture zone of the ACLC. Magnification: 400× (**a**), 200× (**b**, **c**); scale bars: 20 μm (**a**), 50 μm (**b**, **c**)

In group 1, the RZ showed disorganized and kinked collagen fibers (Table 2 and Fig. 7). Shapes of cells and nuclei of fibrocyte-like cells were more spheroid than those in the IZ, indicating cellular metabolic activity. As early as within the first week after trauma, increased numbers of fibroblast-like cells, migrating monocytes, and macrophages were found. These cellularity changes were consistent with an early inflammatory response. In group 2, significant increases of collagen 3 content and fibroblast-like cells were observed. The second week after trauma appeared to be the most proliferative phase showing the highest level of granulation tissue formation. In group 3, the collagen 3 concentration and αSMA-positive cell counts remained high but showed a trend towards decrease when compared to group 2 (*P* > 0.05). Reorganization of the collagen structure and normalization of crimp length indicated consolidation and early remodeling.

**Table 2:** Time-dependent histomorphological changes within the rupture zone

Group (time)	Histomorphological changes within the rupture zone
Group 1 (0–7 days)	Inflammation
	- increase of total cell count and CD68-positive cells
	- metabolic activation of fibrocyte-like cells
Group 2 (8–14 days)	Proliferation, granulation
	- increase of collagen 3 content
	- increase of fibroblast-like cells
Group 3 (15–21 days)	Consolidation, early remodeling
	- reorientation of collagen bundles
	- normalization of crimp length

#### Epiligamentous tissue formation

In group 1, synovial coverage occurred at the distal end of the RZ (Table 3 and Fig. 7a). Small interstitial streaks of granulation tissue were formed along with disrupted collagen fiber bundles. In group 2, an inflammatory response, progressive formation of granulation tissue, and strong neovascularization were observed. A notable increase in fibroblast-like cells and activated αSMA-positive myofibroblasts was also observed (Fig. 7b). Consistent with the histomorphological changes observed in the RZ, the most proliferative phase occurred during the second week after trauma. In group 3, IHC showed infiltrative granulation of the RZ and strong neovascularization of the newly formed clot of granulation tissue (Fig. 7b).

**Table 3:** Time-dependent characteristics of epiligamentous tissue formation

Group (time)	Characteristics of epiligamentous tissue formation
Group 1 (0–7 days)	Synovialization
	- synovial coverage of rupture end
	- streaky formation of granulation tissue
Group 2 (8–14 days)	Inflammation and epiligamentous tissue formation
	- increase of αSMA-positive and fibroblast-like cells
	- strong expression of collagen 3
	- strong formation of the granulation tissue
	- full synovial layer of epiligamentous tissue > 10 days
Group 3 (15–21 days)	Infiltrative granulation, neovascularization
	- neovascularization of newly formed granulation tissue

*αSMA* Alpha-smooth muscle actin

## Discussion

The present study sought to investigate time-dependent healing responses of the human acromioclavicular ligament following acute traumatic rupture. Outcome reveals beneficial intrinsic healing capabilities of the human ACLC during the first week after trauma. This work is significant as it substantially influences the clinical management of ACJ dislocations.

Early surgical therapy of acute ACJ appears to be favorable if surgical treatment is considered. Histologically, only the first week after trauma can be regarded as the acute inflammatory phase. Reparative processes (proliferation and collagen 3 synthesis) already peak within the second posttraumatic week suggesting limited healing potential. Beginning from the third week after trauma, the biological healing potential might be insufficient, as early processes of ligamentous remodeling become evident. Processes of remodeling and proliferative granulation of the rupture ends were consistently found in all samples 2 weeks after trauma. Consequently, biological augmentation with autologous or allogenous tendon grafts may be favorable after the second posttraumatic week, if surgical treatment is indicated. At our institution, conservative treatment is conducted in patients presenting more than 3 weeks after trauma. Chronic ACJ reconstruction using an autologous hamstring tendon graft is then performed if conservative treatment fails. However, prospective clinical studies are needed to evaluate whether early ACJ stabilization truly translates into clinical benefits. Furthermore, cut-off time points need to be defined for biological augmentation. Moreover, additional histological and biomechanical analyses should be performed to gain deeper insights into ACLC tendon graft remodeling.

Ligament healing follows sequential phases of cellular processes such as inflammation, proliferation, and ultimately remodeling [5, 13] In contrast to bradytrophic tendons, ligaments show a more dynamic and pronounced biological response to acute injuries as recently demonstrated for the ACL and MCL of the human knee joint [24, 28–30]. The present findings indicate that the human ACLC exhibits early and highly dynamic intrinsic responses to traumatic rupture. Early cellular changes comprise activation of fibroblasts and invasion of inflammatory cells. Chemotaxis of macrophages with a marked increase of CD68 expression was observed within the first week after trauma. In the second posttraumatic week, the maximum peaks of αSMA expression and collagen type 3 were observed indicating high proliferative activity and scar tissue formation. Moreover, the synovial microenvironment of a full-thickness ACLC tear induced a specific and redundant pattern of healing as observed in the epiligamentous tissue formation at the rupture site. Within the first week after trauma, synovial granulation tissue consistently covered the rupture end. In the second week, this ‘epiligament’ presented infiltrative growth, strong neovascularization, and high rates of αSMA expression. As previously proven for midsubstance ACL tears, αSMA-positive myofibroblasts might cause retraction and progressive dehiscence of the rupture ends, if operative reduction and stabilization do not occur promptly [23, 24]. Both pathomechanisms, excessive scar tissue, and epiligamentous tissue formation, might prevent optimal ligamentous healing and promote insufficient scar healing within the second posttraumatic week. These findings strongly suggest that surgical treatment of acute ACLC tears should be performed within the first posttraumatic week to exploit the utmost intrinsic ligamentous healing capabilities.

Clinical findings on ACLC healing are limited to one MRI-based study displaying hypertrophic consolidation in all 37 cases that were surgically treated after a mean time interval of 8.4 days after injury [31]. To our best knowledge, there is currently no understanding of the intrinsic healing mechanism of human ACLC. Anatomically, the ACLC represents a ligamentous augmentation of the superior ACJ capsule and therefore may be considered as a periarticular ligament [32]. In cases of complete traumatic rupture, the ACLC is exposed to an intraarticular, synovial microenvironment. Thus, comparisons to other human ligaments, particularly around the knee joint, might be helpful for the understanding of ACJ injuries. Past studies have extensively investigated the healing characteristics of the ACL and MCL. In 1999, Lo et al. [33] described the fundamental ability of the human ACL to heal. The authors observed scarred reattachment of tibial ACL remnants onto the posterior cruciate ligament, a mechanism clinically known as ‘lambda’ healing. More recently, Nguyen and coworkers [24] performed detailed histological investigations of spontaneously healed human ACL remnants and found specific mechanisms of intrinsic healing such as neovascularization and an increase in αSMA-positive myofibroblasts as well as collagen type 3 expression. Menetrey et al. [22] compared healing characteristics of partial midsubstance ACL tears with complete, midsubstance (mop-end) MCL tears in a canine knee model. The authors found higher rates of αSMA-positive cells and TGF-1 expression in MCL than ACL tears, suggesting the superior healing potential of the MCL compared to the ACL. As early as 3 days after trauma, a significant invasion of αSMA-positive cells could be detected at the MCL rupture zone. Both, αSMA-induced activation of fibroblasts and synthesis of collagen type 3, are known as essential components of ligamentous healing [34, 35]. Our findings confirm the basic principles of ligament healing described in previous work.

Biomechanically insufficient ACLC healing is a major cause of symptomatic DHI following surgical treatment of acute ACJ dislocations. Current evidence suggests delayed surgery to be a risk factor for impaired ACLC healing. Considering our present findings, we assume that the ideal timeframe for surgery might be closer than previously expected. Furthermore, the dynamics and characteristics of human ACLC healing indicate that the earliest possible surgical intervention appears most beneficial in acute high-grade ACJ dislocations. In vivo, especially the first week after trauma may be regarded as favorable time of surgical therapy, as the acute phase is characterized by inflammatory processes and cellular activation without the proliferation of extracellular matrix components. In contrast, proliferative processes including collagen type 3 production already peaked within the second posttraumatic week. Within the third week (group 3), ligamentous remodeling and a decrease of collagen type 3 expression were apparent suggesting insufficient intrinsic healing capacity of the ACLC. This experimental study further confirms the findings introduced by Barth et al. showing superior results when surgery was performed within a timeframe of 10 days after acute ACJ dislocation [6]. Hence, we support the authors’ conclusion that surgery should be performed within the early inflammatory phase before cell repair mechanisms are initiated.

Our study is associated with strengths and limitations. Major limitations are the small sample size and lack of a healthy control group. An a priori power analysis was not feasible due to the study design and protocol. Though, previous studies on human ligament healing had comparable or even smaller sample sizes [22, 23]. For ethical reasons, biopsies of healthy controls could not be obtained, and group sizes had to be kept as small as possible. The human in vivo model represents both, a strength and a limitation. It is the only experimental model that allows for clinically relevant conclusions on human ACLC healing. On the other hand, patient-specific parameters such as sex, age, ACLC tear type, and individual variations of healing potential might have influenced our outcome. However, strict inclusion and exclusion criteria led to a homogeneous study population minimizing selection bias. Due to methodological reasons, the coracoclavicular ligament complex could not be evaluated in this study.

## Conclusions

The acromioclavicular ligament exhibits early and dynamic healing responses following acute traumatic rupture. Our histological findings suggest that surgical treatment of acute ACJ dislocations should be performed as early as possible within a timeframe of 1 week after trauma to exploit the utmost biological healing potential. Prospective clinical studies are warranted to investigate whether early surgical treatment of ACJ dislocations translates into clinical benefits.

## Data Availability

The datasets used and/or analysed during the current study are available from the corresponding author on reasonable request.

## Abbreviations

ACJ: Acromioclavicular joint
ACLC: Acromioclavicular ligament complex
ACL: Anterior cruciate ligament
αSMA: Alpha-smooth muscle actin
DHI: Dynamic horizontal instability
HPF: High power field
IHC: Immunohistochemistry
IZ: Intact zone
MCL: Medial collateral ligament
NAR: Nuclear aspect ratio
RZ: Rupture zone
SD: Standard deviation
